# Attentional bias in competitive situations: winner does not take all

**DOI:** 10.3389/fpsyg.2015.01469

**Published:** 2015-09-25

**Authors:** Zhongqiang Sun, Tian Bai, Wenjun Yu, Jifan Zhou, Meng Zhang, Mowei Shen

**Affiliations:** Department of Psychology and Behavioral Sciences, Zhejiang University, HangzhouChina

**Keywords:** competition, winner, loser, attentional bias, evolutionary psychology

## Abstract

Compared to previous studies of competition with participants’ direct involvement, the current study for the first time investigated the influence of competitive outcomes on attentional bias from a perspective of an onlooker. Two simple games were employed: the Rock-Paper-Scissors game (Experiment 1) in which the outcome is based on luck, and Arm-wrestling (Experiment 2), in which the outcome is based on the competitors’ strength. After observing one of these games, participants were asked to judge a stimulus presented on either the winner’s or loser’s side of a screen. Both experiments yielded the same results, indicating that the onlookers made much quicker judgments on stimuli presented on the loser’s side than the winner’s side. This suggests the existence of an attention bias for loser-related information once a competition has ended. Our findings provide a new lens through which the influence of competition results on human cognitive processing can be understood.

Some battles you win, some battles you lose.

– The Romance of the Three Kingdoms

Competition is a ubiquitous and age-old behavior pattern and can range from a rivalry between two contestants to a war among several tribes. As the opening quotation suggests, competition is cruel because victory and defeat always come along with it. With regard to the influence of competition on the surrounding, the outcome of the rivalry may be the most important aspect. For instance, in social context, victory or defeat in war could potentially determine the survival of a tribe; while in dyadic context, an individual’s win or lose in a competition could also affect the way of being treated by other people. There’s an old saying that winner takes all. Is it also true in the social-cognitive processes? How would the asymmetric competing outcome direct the third-party onlooker’s early stage processing on winner-/loser-related information? The answer still remains unclear, and is what we concerned in the current research.

Psychological research has valued the study of competition for decades. Compared to the research from other multiple disciplines, including sociology (e.g., Axelrod, 1997; Podolny, 2010), organizational behavior (Malhotra, 2010), education (Conti et al., 2001), and even biology and ecology (Earley et al., 2013), psychological studies pay more attention to information processing and behavioral patterns during the competitive interaction, as well as the influence of competition on subsequent interactions with others in the social group. Most studies have focused on the interaction during the competitive process, in which competition has been found to affect the actions of the moment (Ruys and Aarts, 2010) and the judgment and evaluation of others (Vonk, 1998), and to even distort cognitive representations (Xiao et al., 2012). In addition, compared to cooperation, competition is different in terms of both individual action patterns and neural activation (Decety et al., 2004; Georgiou et al., 2007).

As a shared experience, the competitive scenario in society consists of three components: the competitor, the competitive process, and the effects of the outcome on others in the social group. The first two components are determined by the competitors themselves, and the latter one is determined by the third-party onlookers. These two types of people may experience very different cognitive processes, so a full picture of the competition event requires an integration of these processes. However, previous research has focused on the former two components, resulting in an insufficient understanding of the process from the perspective of the third-party onlookers.

The existing findings related to the third-party perspective, however, limited, mainly concern the high-level conscious processing of logical reasoning and moral judgment, especially with regard to third-party punishment, which concerns how non-stakeholders punish the offender during the competition (e.g., Fehr and Gächter, 2002; Fehr and Fischbacher, 2004).

Although such mechanisms underlying high-order processes have been examined by empirical studies, in terms of evolutionary theories, adaptive psychological mechanisms are presumed to exist at all levels of cognition, including both the aforementioned high-order processes and the relatively automatic early stage forms, such as attention and perception. Early stage cognition is of equal importance to the high-order process because those underlying mechanisms are the cornerstones shaping adaptive high-order cognition (see Kurzban et al., 2001; Maner et al., 2007); however, this area has been left relatively unexplored.

Therefore, the current research aimed to fill this gap in the literature by placing an emphasis on how the competitive outcomes influence the third-party onlookers in terms of the distribution of early stage attentional resources. In particular, by displaying a competitive interaction to an individual, we investigated the shifting of attention immediately after the outcome being announced. As we know, attention is the door to human cognition, and all unequal distributions of our cognitive resources originate from attention. It is also a key to enable us to understand this world by selecting relevant information out of irrelevant noise and processing the important parts of the information we receive (Carrasco, 2011). In this specific competitive situation, the outcomes were always asymmetric (i.e., not a draw). The side (winner or loser) with higher subjective value should capture more visual attention, and the information relevant to the winner and loser would then be processed differently from the perspective of the third-party onlooker.

We hypothesized that the loser, as a kind of negative stimulus, would capture attention first. It is evolutionarily adaptive for negative information to be more influential than positive (Baumeister et al., 2001) because negative things may threaten one’s survival. This advantage in terms of processing negative stimuli has been extensively demonstrated from multiple aspects. For instance, compared to positive stimuli, negative stimuli capture attention earlier (Eastwood et al., 2001; Fox et al., 2002; Koster et al., 2004; Soares et al., 2009), are memorized more solidly (Taylor, 1991), and are constructed with more cognitive interpretations (Abele, 1985).

In social interactions particularly, cooperating with each other is an effective way to help individuals increasing their fitness. It can be risky, though, since the chance of survival also depends largely on how the individuals choose their partner. If a loser who is an incapable partner is chosen, the strength of the group will be heavily discounted, which may further hinder the achievement of group success. In this sense, the strategy of cooperating without considering the capability and history of one’s partners is not optimal in the long run. Instead, a more egoistic strategy would enable an individual to detect and subsequently avoid a loser.

Similar mechanisms have been suggested in cheater detection studies (Cosmides and Tooby, 1989; Tooby and Cosmides, 2005). Research shows an enhanced memory for untrustworthy faces rather than for trustworthy faces, revealing that untrustworthy faces were of high ecological value and relatively salient (e.g., Mealey et al., 1996; Oda, 1997; Yamagishi et al., 2003; Bayliss and Tipper, 2006). This mechanism of human bias in information processing may exist not only for untrustworthy individuals in social exchange, but also for a range of other harmful stimuli (Bell and Buchner, 2012). Given the reviewed empirical studies, we predicted an attentional bias toward loser-related information.

To investigate the influence of a competition situation, it is true that a naturalistic context or paradigm would be ideal, but the results would be affected by too many uncontrolled factors simultaneously. For instance, the winner and loser are likely to show different expressions and behaviors at the conclusion of an agonistic encounter (Lippold et al., 2008; Matsumoto and Hwang, 2012), which would affect the onlooker’s attention distribution to a large extent. Fortunately, it is possible to control for these factors in the context of a laboratory experiment. Our paradigm was to display to participants two kinds of competitive games on a computer screen to represent the competition situation. In this way, we excluded the personal features of the competitors and isolated the winning/losing information, enabling control of detection of onlooker’s rapid switching of attention. In Experiment 1, the Rock-Paper-Scissors game (RPS) was presented as the competitive situation. As a popular and simple game, it is widely used to study competition-related issues (e.g., Sinervo and Lively, 1996; Semmann et al., 2003; Wang et al., 2014). This game has a strong advantage in that three candidate actions are mutually restricted, and no action holds absolute predominance: Rock defeats Scissors, Scissors defeat Paper, and Paper defeats Rock. To be specific, each gesture could be either the winner or the loser in different pair condition, which could be regarded as a counterbalance procedure. By synthesizing all three pairs conditions in our analysis, the influence of visual difference of various stimuli could be minimized. However, the outcomes of the RPS game are considered to be based on luck to a large extent. Given that most match results are based on competitors’ different capabilities, we hence adopted in Experiment 2 another popular game, arm-wrestling, which requires actual strength. Rapid reaction and judgment are necessary to examine automatic early stage mechanisms. Both games employed in the current study are advantageous for this consideration.

## Experiment 1

### Methods

#### Participants

Fourteen participants (seven females, 18–26 years-old) were paid to participate in the experiment. All had no history of neurological problems and had normal or corrected-to-normal vision. The participants provided written and informed consent before the experiments, and the procedures were in compliance with the Code of Ethics of the World Medical Association (Declaration of Helsinki), as well as approved by the Research Ethics Board of Zhejiang University.

The sample size in the current study was determined by a power analysis based on predicted effect size, using G*power 3 (Faul et al., 2007, 2009). According to the effect size ($\eta_{p}^{2}$ = 0.22) obtained from the pilot experiment, the analysis suggested a sample size of 14. This sample size was adopted in all the following experiments.

#### Stimuli

**FIGURE 1:**
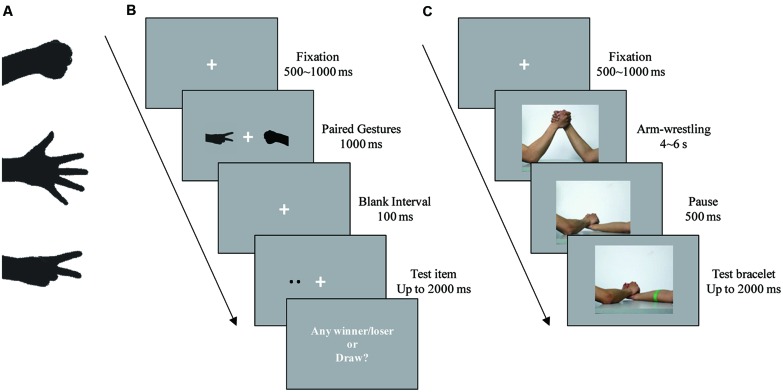
**Stimulus and procedures in Experiments 1 and 2. (A)** Three gestures used in Experiment 1. From top to bottom, the gestures are Rock, Paper, and Scissors. **(B)** An example of a trial in Experiment 1 with two dots on the loser’s side as the test item. **(C)** An example of a trial in Experiment 2 with a green bracelet on the loser’s arm as the test item.

Three pictures of gestures were adopted from the RPS game (see **Figure 1A**). In order to eliminate the influence of luminance difference, the gesture pictures were monochromatized to black (0, 0, 0, RGB). Stimuli were presented on a gray background (80, 80, 80) CRT monitor of a 17-inch computer (100 Hz refresh rate). Each gesture occupied a 3° × 4° rectangular area, centered 5° to the left or right of a central fixation cross. The direction of each gesture horizontally pointed to either left or right in different experimental conditions. Two or three dots were set as the test item.

#### Design and Procedure

Participants were seated in an electrically shielded and sound-attenuated recording chamber at a distance of 70 cm from the CRT monitor. Participants were asked to keep their eyes centrally fixated.

The procedure of Experiment 1 is shown in **Figure 1B**. We designed a dual-task paradigm, which was revised from Posner’s cueing paradigm (Posner, 1980). Each trial began with a fixation cross presented randomly for a duration of 500 to 1000 ms; then, a gesture array was displayed for 1000 ms, consisting of two same or different gestures. When two different gestures were displayed, the winner and loser were determined (i.e., Rock defeats Scissors, Scissors defeats Paper, and Paper defeats Rock); otherwise, a draw was declared. Then, a 100-ms blank interval was inserted, followed by a 2000-ms test item. The test item was located either at the same position as the winner (Test-in-Winner condition) in 50% of the non-draw trials or the same position as the loser (Test-in-Loser condition) in the rest of the non-draw trials. The position of the winning gesture was balanced between left and right. If it was a draw, the test item was randomly located in either the left or the right visual field. The participant was first required to indicate whether the test item contained two dots or three dots by pressing one of two keys, with accuracy rather than response speed being stressed. Then, after a 500-ms blank interval, a secondary task required the participant to recall whether the Paired Gesture was a draw and respond by pressing one of another two keys. This secondary task was presented to keep the participants involved when seeing the gesture array. The interval between trials was randomly set from 1000 to 1500 ms (see detailed videos on the website: http://www.psych.zju.edu.cn/english/redir.php?catalog_id=15773).

Each participant completed 48 trials for each of the two test-item positions (Test-in-Winner and Test-in-Loser), which were evenly distributed among the three possible winner-loser situations (Rock-Scissors, Scissors-Paper, and Paper-Rock). They completed another 48 trials for the draw condition, resulting in a total of 144 randomly presented trials. The whole experiment was divided into three blocks with a 2-min break between blocks. Before the formal experiment, there were at least 20 practice trials to ensure that the participants understood the instructions.

### Results

Trials with inaccurate responses were excluded from the reaction time (RT) analyses (7.19% of all trials), as well as the outliers with RTs more than 2 SD above or below the mean (4.81% of all trials).

To exclude the potential influence of unilateral advantage, we compared the RTs in the draw condition between the situations when the dot was displayed in the left and right visual fields, and no significant difference was found (left, mean ±*SD*, 794.92 ± 119.39 ms; right, 805.93 ± 115.56), *t*(13) = -0.81, *p* > 0.250. Given that draws were not of interest to us, we will not discuss draw outcomes in the following sections.

We conducted a two-way analysis of variance (ANOVA) for dual-task RT and accuracy, with test-item position (Test-in-Winner and Test-in-Loser) and winner-loser situations (Rock-Scissors, Scissors-Paper, and Paper-Rock) as independent variables for non-draw data.

**FIGURE 2:**
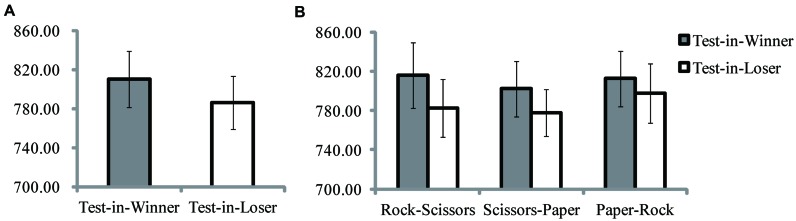
**Results of Experiment 1. (A)** Reaction times (ms) for Test-in-Winner and Test-in-Loser conditions. **(B)** Reaction times (ms) for Test-in-Winner and Test-in-Loser conditions with three paired gestures. The error bars represent one SEM.

Interestingly, for RT, a significant main effect for test-item position was found, *F*(1,13) = 7.03, *p* = 0.020, $\eta_{p}^{2}$ = 0.35, while none was found for winner-loser situation (see **Figure 2A**), *F*(2,26) = 1.08, *p* > 0.250, $\eta_{p}^{2}$ = 0.08. *Post hoc* contrast analyses revealed a somewhat faster response speed for items on the loser side [784.83 ± 101.25, 95% Confidence Interval or 95% CI (727.53, 842.13)] compared to the winner side [811.34 ± 108.61, 95% CI (749.05, 873.63)]. Moreover, no interaction was found between test-item position and winner-loser situation, *F*(2,26) = 0.93, *p* > 0.250, $\eta_{p}^{2}$ = 0.07, implying that performance with all three winner-loser situations shared almost the same tendency in terms of results (see **Figure 2B**)^1^. No main effect for accuracy was found for either test-item position [Test-in-Winner, 93.06 ± 3.89%, 95% CI (90.81%, 95.30%)]; [Test-in-Loser, 92.56 ± 3.68%, 95% CI (90.44%, 94.68%)], *F*(1,13) = 0.28, *p* > 0.250, $\eta_{p}^{2}$ = 0.02, or winner-loser situation, *F*(2,26) = 0.08, *p* > 0.250, $\eta_{p}^{2}$ = 0.01, nor was there interaction between the variables, *F*(2,26) = 1.23, *p* > 0.250, $\eta_{p}^{2}$ = 0.09. The accuracy results strongly confirmed that the salient RT difference was not due to a speed-accuracy trade-off.

## Experiment 2

In Experiment 1, we found that the attention of the participants, as third-party onlookers, was captured by the information on the loser’s side. One might argue that the gestures appearing in the RPS game are randomly chosen by the competitors and presented to the onlookers, and thus the outcomes are based on luck to a large extent. As we know, most competition outcomes are not the result of luck but are directly relevant to a competitor’s actual ability, such as strength, or power; that is to say, the strongest wins. Therefore, it is of great importance to verify the above results in a situation that depends on a competitor’s strength. Accordingly, in Experiment 2, we adopted another traditional type of match: arm-wrestling. During the match, two competitors hold their left or right hands together and try their best to press and move the other’s hand. The one who presses the other’s hand onto the table first is determined to be the winner. Unlike the RPS game, a draw cannot be declared because the winner and loser are always decided for each round.

### Methods

#### Participants

A new group of 14 participants (five females, 19–26 years-old) was paid to participate in the experiment. Other aspects were the same as those in Experiment 1.

#### Stimuli

The arm-wrestling match between two volunteers (S & W) was recorded by a camera without showing any identifying information such as the face and clothes. Since the match itself is quite simple, participants may become accustomed to the video information after several trials and respond before the result comes out when they see the first part of the video. To prevent this occurrence, three different situations were adopted to play the match out when either S or W was the winner. The following are three situations that could apply when S is the winner:

Easy-win: after a 3–4 s stalemate, S wins; total match duration of 4 s;
Hard-win: S plays more strongly than W at the beginning, and after a 1–2 s stalemate, S wins; total match duration of 5 s;
Super-hard-win: W plays more strongly than S at the beginning, and after a 1–2 s stalemate, S fights back to win; total match duration of 6 s.

The three same situations were applied when W was the winner. Those six videos were also processed to create another six mirrored versions by exchanging the position of the two volunteers in order to balance the position of the winner. All 12 videos were presented in a mixed order during the experiment. Each video occupied a 20° × 20° rectangular area on a gray background (80, 80, 80) of a 17-inch CRT monitor (100 Hz refresh rate).

In addition, we froze the last frame of the video in which the winner/loser had just been declared for use as the test picture, and we attached an extra red or green bracelet to the arm of one volunteer as the test item.

#### Design and Procedure

The procedure for Experiment 2 is shown in **Figure 1C**. After presentation of a fixation cross lasting 500–1000 ms, one of the videos was shown in the center of screen. Once the winner/loser was declared, the video paused for 500 ms, followed by a 2000-ms presentation of the test picture. In the test picture, the red or green bracelet was located on the winner’s arm in 50% of the trials (Test-in-Winner condition) or the loser’s in the rest of the trials (Test-in-Loser condition). The participant was required to judge the color of the bracelet, with accuracy rather than response speed being stressed. The interval between trials was randomly set from 1000 to 1500 ms.

Each participant completed 96 trials for each of the two conditions, with a total of 192 randomly presented trials. These trials were evenly distributed among the 12 aforementioned videos. The whole experiment was divided into four blocks with a 2-min break between blocks. Before the formal experiment, there were at least 20 trials for practice to ensure that the participants understood the instructions.

### Results

Trials either with inaccurate responses (2.49% of all trials) or with RTs more than 2 SD above or below the mean (3.42% of all trials) were excluded from the RT analyses.

Similar to Experiment 1, two-way ANOVAs were conducted for both RT and accuracy, with test-item position (Test-in-Winner and Test-in-Loser) and match situation (Easy-win, Hard-win, and Super-hard-win) as independent variables.

**FIGURE 3:**
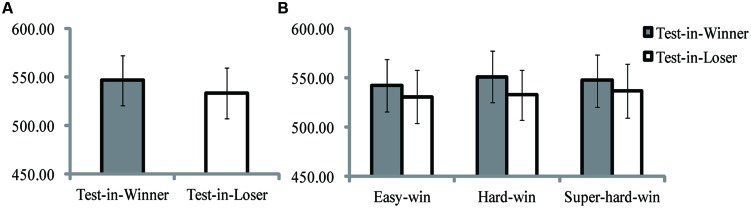
**Results of Experiment 2. (A)** Reaction times (ms) of Test-in-Winner and Test-in-Loser conditions. **(B)** Reaction times (ms) of Test-in-Winner and Test-in-Loser conditions in three match situations. The error bars represent one SEM.

The results for RT almost replicated those in Experiment 1. A significant main effect was only found for test-item position (see **Figure 3A**), *F*(1,13) = 10.27, *p* = 0.007, $\eta_{p}^{2}$ = 0.44. *Post hoc* contrast showed a relatively shorter RT in the Test-in-Loser condition [533.58 ± 98.28, 95% CI (477.78, 591.28)] than in Test-in-Winner [546.87 ± 96.45, 95% CI (491.74, 603.39)]. Nor significant main effect for match situation, *F*(2,26) = 1.13, *p* > 0.250, $\eta_{p}^{2}$ = 0.08, nor interaction was found (see **Figure 3B**), *F*(2,26) = 0.49, *p* > 0.250, $\eta_{p}^{2}$ = 0.04.

For accuracy, the only significant main effect was found for test item position, *F*(1,13) = 6.01, *p* = 0.030, $\eta_{p}^{2}$ = 0.32, while the main effect for match situation was not significant, *F*(2,26) = 1.68, *p* = 0.210, $\eta_{p}^{2}$ = 0.11. There was also no interaction between test-item position and match situation, *F*(2,26) = 0.05, *p* > 0.250, $\eta_{p}^{2}$ = 0.004. *Post hoc* analysis revealed a slightly higher accuracy when the test item was on the loser side [98.09 ± 1.51%, 95% CI (97.22%, 98.96%)] rather than on the winner side [96.97 ± 2.39%, 95% CI (95.60%, 98.35%)]. The accuracy results again excluded the potential influence of a speed-accuracy trade-off.

## Discussion

The results indicated that the third-party onlookers made quicker judgments for stimuli presented on the loser’s side compared to those on the winner’s side, implying the existence of an attentional bias toward the loser. Two competitive games were included, based either on the competitors’ ability or on random chance. In Experiment 1, in which the competitor throws rock, paper, or scissors randomly, the onlookers responded to stimuli presented on the loser’s side much more quickly, though these stimuli were not directly relevant to the loser. In Experiment 2, we presented an arm-wrestling game, a competition that required strength on the part of the competitor, and we attached a colored bracelet to the target competitor’s arm. The results suggested a faster response for loser-related information, which replicated the pattern in the RPS game. The findings here demonstrated that no matter whether the competition outcome was decided randomly or with real strength, the onlookers vigilantly attended to stimuli that were relevant to the loser.

This attentional bias toward the loser in the competition was thus verified for the first time from the perspective of a third-party onlooker. The unequal information (i.e., winner and loser) that is generated from social interaction behaviors such as competition leads to a bias in third-party onlookers’ early information processing. This fits with the theory that humans vigilantly attend to negative information, which is known as negativity bias. This bias focuses on the adaptive implications of negative-positive asymmetrical processes with the result that negative events are more salient and dominant in many situations (Taylor, 1991; Cacioppo and Berntson, 1994; Rozin and Royzman, 2001). If a negative stimulus is overlooked, people could lose some portion of their own resources, or even worse, pay the price of losing their life and decreasing the possibility of perpetuating their genes (Baumeister et al., 2001). In competitive situations, the loser represents this negative stimulus. Hence, it is reasonable to process this negative stimulus more quickly and accurately than neutral or positive stimuli, as this may result in an increased chance of survival.

Quickly detecting losers is not only a more egoistic strategy for individuals, but it is also a stable strategy for group survival. This can be analyzed using evolutionary game theory (Smith, 1982). If a strategy adopted by a population guides interactions and persists in the group for a long time because it produces more fitness benefit and outweighs any alternative strategy, it is known as an evolutionary stable strategy (ESS; Smith, 1982). In our study, it could be interpreted as a necessary condition to reliably detect the loser in a human interaction. For example, if an individual cannot reliably detect a loser, their unconditional collaborating with the loser will increase the fitness of any loser they meet in the population. When cooperating with a loser, however, his/her low probability of success will lead to an unrewarding cooperation, as well as a net fitness cost. As a result, a population of unconditional collaborators could be invaded and finally outcompeted due to their using this behavioral strategy with a lower probability of success, when compared to those who avoid losers and seeks winners with whom to cooperate. In this case, conditional cooperation, which requires the ability to detect losers, is an ESS.

Moving beyond previous studies in which participants were involved in competition as a contestant, the current research was instead conducted from the perspective of a third-party onlooker. Additionally, the complex competitive behaviors of humans were represented here by two simple and classical games, the RPSs and Arm-wrestling, that could be manipulated easily in behavioral studies, thus providing a novel opportunity to investigate the current issue.

Furthermore, there might exist some interesting issues coming along with the current finding. Apart from attention, does the asymmetric competing outcome also affect human’s other cognitive processing such as perception and memory? For instance, a mnemonic advantage was already found on cheater-related information (Bell and Buchner, 2012). Analogically, is it possible for loser to induce a similar mnemonic bias toward itself? Further studies need to examine the specific mechanism causing this attentional bias and extend its application. For instance, previous studies found that attention bias modification procedure could reduce attentional bias for threat, thereby diminishing anxiety symptom (e.g., MacLeod et al., 2002; Heeren et al., 2015; Linetzky et al., 2015). Therefore, it could be possible that experimental training inducing an attention bias toward gain-related material will modify competitive information vulnerability, which may decease the attentional bias toward loser-related information. Meanwhile, it is also intriguing to explore that whether this attention bias is innate from one’s birth or acquired from social interaction experience later in life. Appropriate adjustment on current paradigm might benefit to find out its answer in children of different ages.

## Conclusion

As the first and common doorway of cognition, attention helps us determine which information takes priority to be encoded. Further processing, such as logical reasoning or decision making, can only be accessed once the information has been attended to. The findings from these two experiments suggest an attentional bias toward loser-related information in a competitive situation. The current research advances the social study concerning competition, and develops the extent of studying the influence of this social interaction on our cognitive function, to an early stage processing level.

## Conflict of Interest Statement

The authors declare that the research was conducted in the absence of any commercial or financial relationships that could be construed as a potential conflict of interest.

## Footnotes

1 We also converted all RT data (Experiments 1 and 2) by logarithmic transformation (Ln), and re-analyzed those transformed data in both two experiments. The patterns of the results after logarithmic transformation were the same as the original statistical analysse.

## References

[B1] Abele A. (1985). Thinking about thinking: causal, evaluative and finalistic cognitions about social situations. *Eur. J. Soc. Psychol.* 15 315–332. 10.1002/ejsp.2420150306

[B2] Axelrod R. M. (1997). *The Complexity of Cooperation: Agent-Based Models of Competition and Collaboration.* Princeton, NJ: Princeton University Press.

[B3] Baumeister R. F. Bratslavsky E. Finkenauer C. Vohs K. D. (2001). Bad is stronger than good. *Rev. Gen. Psychol.* 5 323–370. 10.1037/1089-2680.5.4.323

[B4] Bayliss A. P. Tipper S. P. (2006). Predictive gaze cues and personality judgments should eye trust you? *Psychol. Sci.* 17 514–520. 10.1111/j.1467-9280.2006.01737.x 16771802 PMC2080823

[B5] Bell R. Buchner A. (2012). How adaptive is memory for cheaters? *Curr. Dir. Psychol. Sci.* 21 403–408. 10.1177/0963721412458525

[B6] Cacioppo J. T. Berntson G. G. (1994). Relationship between attitudes and evaluative space: a critical review, with emphasis on the separability of positive and negative substrates. *Psychol. Bull.* 115 401–423. 10.1037/0033-2909.115.3.401

[B7] Carrasco M. (2011). Visual attention: the past 25 years. *Vision Res.* 51 1484–1525. 10.1016/j.visres.2011.04.012 21549742 PMC3390154

[B8] Conti R. Ann M. Picariello M. L. (2001). The impact of competition on intrinsic motivation and creativity: considering gender, gender segregation and gender role orientation. *Pers. Individ. Differ.* 31 1273–1289. 10.1016/S0191-8869(00)00217-8

[B9] Cosmides L. Tooby J. (1989). Evolutionary psychology and the generation of culture, part II: case study: a computational theory of social exchange. *Ethol. Sociobiol.* 10 51–97. 10.1016/0162-3095(89)90013-7

[B10] Decety J. Jackson P. L. Sommerville J. A. Chaminade T. Meltzoff A. N. (2004). The neural bases of cooperation and competition: an fMRI investigation. *Neuroimage* 23 744–751. 10.1016/j.neuroimage.2004.05.025 15488424 PMC3640982

[B11] Earley R. L. Lu C. Lee I. Wong S. C. Hsu Y. (2013). Winner and loser effects are modulated by hormonal states. *Front. Zool.* 10:6 10.1186/1742-9994-10-6 PMC3598835 23399457

[B12] Eastwood J. D. Smilek D. Merikle P. M. (2001). Differential attentional guidance by unattended faces expressing positive and negative emotion. *Percept. Psychophys.* 63 1004–1013. 10.3758/BF03194519 11578045

[B13] Faul F. Erdfelder E. Buchner A. Lang A. G. (2009). Statistical power analyses using G*Power 3.1: tests for correlation and regression analyses. *Behav. Res. Methods* 41 1149–1160. 10.3758/BRM.41.4.1149 19897823

[B14] Faul F. Erdfelder E. Lang A. G. Buchner A. (2007). G*Power 3: a flexible statistical power analysis program for the social, behavioral, and biomedical sciences. *Behav. Res. Methods* 39 175–191. 10.3758/BF03193146 17695343

[B15] Fehr E. Fischbacher U. (2004). Third-party punishment and social norms. *Evol. Hum. Behav.* 25 63–87. 10.1016/S1090-5138(04)00005-4

[B16] Fehr E. Gächter S. (2002). Altruistic punishment in humans. *Nature* 415 137–140. 10.1038/415137a 11805825

[B17] Fox E. Russo R. Dutton K. (2002). Attentional bias for threat: evidence for delayed disengagement from emotional faces. *Cogn. Emot.* 16 355–379. 10.1080/02699930143000527 18273395 PMC2241753

[B18] Georgiou I. Becchio C. Glover S. Castiello U. (2007). Different action patterns for cooperative and competitive behaviour. *Cognition* 102 415–433. 10.1016/j.cognition.2006.01.008 16516188

[B19] Heeren A. Mogoase C. McNally R. J. Schmitz A. Philippot P. (2015). Does attention bias modification improve attentional control? A double-blind randomized experiment with individuals with social anxiety disorder. *J. Anxiety Disord.* 29 35–42. 10.1016/j.janxdis.2014.10.007 25465885

[B20] Koster E. H. Crombez G. Van Damme S. Verschuere B. De Houwer J. (2004). Does imminent threat capture and hold attention? *Emotion* 4 312–317. 10.1037/1528-3542.4.3.312 15456400

[B21] Kurzban R. Tooby J. Cosmides L. (2001). Can race be erased? Coalitional computation and social categorization. *Proc. Natl. Acad. Sci. U.S.A.* 98 15387–15392. 10.1073/pnas.251541498 11742078 PMC65039

[B22] Linetzky M. Pergamin-Hight L. Pine D. S. Bar-Haim Y. (2015). Quantitative evaluation of the clinical efficacy of attention bias modification treatment for anxiety disorders. *Depress. Anxiety* 32 383–391. 10.1002/da.22344 25708991

[B23] Lippold S. Fitzsimmons L. P. Foote J. R. Ratcliffe L. M. Mennill D. J. (2008). Post-contest behaviour in black-capped chickadees (*Poecile atricapillus*): loser displays, not victory displays, follow asymmetrical countersinging exchanges. *Acta Ethol.* 11 67–72. 10.1007/s10211-008-0043-4

[B24] MacLeod C. Rutherford E. Campbell L. Ebsworthy G. Holker L. (2002). Selective attention and emotional vulnerability: assessing the causal basis of their association through the experimental manipulation of attentional bias. *J. Abnorm. Psychol.* 111 107–123. 10.1037/0021-843X.111.1.107 11866165

[B25] Malhotra D. (2010). The desire to win: the effects of competitive arousal on motivation and behavior. *Organ. Behav. Hum. Decis. Process.* 111 139–146. 10.1016/j.obhdp.2009.11.005

[B26] Maner J. K. Gailliot M. T. DeWall C. N. (2007). Adaptive attentional attunement: evidence for mating-related perceptual bias. *Evol. Hum. Behav.* 28 28–36. 10.1016/j.evolhumbehav.2006.05.006

[B27] Matsumoto D. Hwang H. S. (2012). Evidence for a nonverbal expression of triumph. *Evol. Hum. Behav.* 33 520–529. 10.1016/j.evolhumbehav.2012.01.005

[B28] Mealey L. Daood C. Krage M. (1996). Enhanced memory for faces of cheaters. *Ethol. Sociobiol.* 17 119–128. 10.1016/0162-3095(95)00131-X

[B29] Oda R. (1997). Biased face recognition in the prisoner’s dilemma game. *Evol. Hum. Behav.* 18 309–315. 10.1016/S1090-5138(97)00014-7

[B30] Podolny J. M. (2010). *Status Signals: A Sociological Study of Market Competition.* Princeton, NJ: Princeton University Press.

[B31] Posner M. I. (1980). Orienting of attention. *Q. J. Exp. Psychol.* 32 3–25. 10.1080/00335558008248231 7367577

[B32] Rozin P. Royzman E. B. (2001). Negativity bias, negativity dominance, and contagion. *Pers. Soc. Psychol. Rev.* 5 296–320. 10.1207/S15327957PSPR0504_2

[B33] Ruys K. Aarts H. (2010). When competition merges people’s behavior: interdependency activates shared action representations. *J. Exp. Soc. Psychol.* 46 1130–1133. 10.1016/j.jesp.2010.05.016

[B34] Semmann D. Krambeck H. J. Milinski M. (2003). Volunteering leads to rock–paper–scissors dynamics in a public goods game. *Nature* 425 390–393. 10.1038/nature01986 14508487

[B35] Sinervo B. Lively C. M. (1996). The rock-paper-scissors game and the evolution of alternative male strategies. *Nature* 380 240–243. 10.1038/380240a0

[B36] Smith J. M. (1982). *Evolution and the Theory of Games.* Cambridge: Cambridge university press 10.1017/CBO9780511806292

[B37] Soares S. C. Esteves F. Flykt A. (2009). Fear, but not fear-relevance, modulates reaction times in visual search with animal distractors. *J. Anxiety Disord.* 23 136–144. 10.1016/j.janxdis.2008.05.002 18565724

[B38] Taylor S. E. (1991). Asymmetrical effects of positive and negative events: the mobilization-minimization hypothesis. *Psychol. Bull.* 110 67–85. 10.1037/0033-2909.110.1.67 1891519

[B39] Tooby J. Cosmides L. (2005). “Conceptual foundations of evolu–tionary psychology,” in *The Handbook of Evolutionary Psychology*, ed. Buss D. M. (Hoboken, NJ: John Wiley & Sons), 5–67.

[B40] Vonk R. (1998). Effects of cooperative and competitive outcome dependency on attention and impression preferences. *J. Exp. Soc. Psychol.* 34 265–288. 10.1006/jesp.1998.1350

[B41] Wang Z. Xu B. Zhou H. H. (2014). Social cycling and conditional responses in the Rock-Paper-Scissors game. *Sci. Rep.* 4 5830 10.1038/srep05830 PMC5376050 25060115

[B42] Xiao Y. J. Van Bavel J. J. Van Bavel J. J. Cunningham W. A. (2012). See your friends close and your enemies closer: social identity and identity threat shape the representation of physical distance. *Pers. Soc. Psychol. Bull.* 38 959–972. 10.1177/0146167212442228 22510363

[B43] Yamagishi T. Tanida S. Mashima R. Shimoma E. Kanazawa S. (2003). You can judge a book by its cover: evidence that cheaters may look different from cooperators. *Evol. Hum. Behav.* 24 290–301. 10.1016/S1090-5138(03)00035-7

